# #PrEP4Love: An Evaluation of a Sex-Positive HIV Prevention Campaign

**DOI:** 10.2196/12822

**Published:** 2019-06-17

**Authors:** Jessica M Dehlin, Ryan Stillwagon, Jim Pickett, Lance Keene, John A Schneider

**Affiliations:** 1 Department of Medicine University of Chicago Chicago, IL United States; 2 Department of Sociology University of British Columbia Vancouver, BC Canada; 3 AIDS Foundation of Chicago Chicago, IL United States; 4 Social Service Administration University of Chicago Chicago, IL United States

**Keywords:** pre-exposure prophylaxis, public health, health equity, health promotion

## Abstract

**Background:**

Pre-exposure prophylaxis (PrEP) is an effective but underutilized method for preventing HIV transmission in communities vulnerable to HIV. Public health campaigns aimed at increasing PrEP awareness and access have less evaluation data.

**Objective:**

The aim of this study was to evaluate Chicago’s PrEP campaign, PrEP4Love (P4L), a campaign that uses health equity and sex-positivity approaches for information dissemination.

**Methods:**

P4L launched in February 2016 and remains an active campaign to date. The analysis period for this paper was from the launch date in February 2016 through May 15, 2016. Our analysis reviews the Web-based reach of the campaign through views on social media platforms (Facebook and Instagram), *smart ads*, or ads served to individuals across a variety of Web platforms based on their demographics and browsing history, and P4L website clicks.

**Results:**

In total, 40,913,560 unique views were generated across various social media platforms. A total of 24,548 users clicked on P4L ads and 32,223,987 views were received from *smart ads*. The 3 most clicked on ads were *STD Signs & Symptoms—More Information on STD Symptoms*, *HIV & AIDS Prevention*, and *HIV Prevention Medication*. An additional 6,970,127 views were gained through Facebook and another 1,719,446 views through Instagram. There was an average of 182 clicks per day on the P4L website.

**Conclusions:**

This is the first study investigating public responses to a health equity and sex-positive social marketing campaign for PrEP. Overall, the campaign reached millions of individuals. More studies of PrEP social marketing are needed to evaluate the relationship of targeted public health campaigns on stigma and to guide future PrEP promotion strategies.

## Introduction

### Background

Chronic illnesses such as HIV or AIDS require renewed public health responses centered around health equity [1] to reduce growing disparities between different racial communities in the United States. In absolute terms, the rate of new HIV infections in the United States has remained stable from 2012 to 2016 among the general population and there were 38,739 diagnoses 2017 [2]. In relative terms, the rates of new HIV infection are on the rise [3] and disproportionately affect gay, black, and Latinx men, where 1 in 2 black, gay men and 1 in 4 gay Latinx men are projected to acquire HIV during their lifetimes [4]. A health equity approach considers how the confluence of upstream, structural oppressions [5], and downstream, individual outcomes, merge to produce widening health disparities among certain populations in the United States. Such populations in Chicago include black, gay and bisexual men and other men who have sex with men (BMSM), transgender women of color, and cisgender women of color [6] who are highly impacted by the HIV epidemic [7]. We used an evidence-based, health equity approach that aims to increase PrEP awareness and adoption among individuals most at risk of HIV infection in Chicago [8].

### Pre-Exposure Prophylaxis as Prevention

Pre-exposure Prophylaxis (PrEP; Tenofovir-Emtricitabine, sold under the brand name Truvada) is an effective biomedical intervention that can decrease the risk of HIV infection by up to 99% [9]. Although PrEP uptake in the United States has increased over the years when the Food and Drug Administration (FDA) approved it in 2012 [10], to date, PrEP adoption among sexual and gender minority communities (eg, racial, gender, and sexual) remains limited [8], most likely because of unaddressed social determinants such as lesbian, gay, bisexual, transgender and queer (LGBTQ) stigma [11], intergenerational poverty, inadequate housing, and high rates of unemployment and incarceration—all of which impact health [12,13]. Furthermore, Gilead, a pharmaceutical company and the only company with FDA approval to manufacture and market PrEP, previously stated that they did not view PrEP as a commercial opportunity and had not participated in commercial advertising [14].

### Grassroots Pre-Exposure Prophylaxis Promotion

PrEP activism amid pharmaceutical and societal indifference is one of many *health social movements* [15] that have thrived through organization and agitation. The aim of PrEP activism in the United States has been to increase knowledge about the rising rates of new HIV infections among black and Latinx individuals, prioritize the issue of health access and treatment among individuals regardless of their social position or financial resources, and give voice to the populations most impacted by the epidemic today in nonstigmatizing, empowering, and relatable ways [16]. Previous public health campaigns have covered topics such as the impact of air pollution on the African American community [17], the health risks of tobacco and vaping among young people [18,19], and teen pregnancy prevention [20]. Within the field of HIV prevention, campaigns have increased condom use among teens [21] and routine HIV testing among women [22].

Where Gilead was absent, the PrEP health social movement was active. Across the globe nonprofit and public health organizations filled the pharmaceutical marketing gap through mass media campaigns designed to increase PrEP awareness, acceptability, and access in the United States [23-26] and around the world [27-30]. PrEP advertising thus emerged as a localized grassroots phenomenon among LGBTQ health and public health organizations in various US and global cities [31]. One of the benefits of PrEP marketing without Gilead was the freedom that various LGBTQ health and public organizations had in tailoring its message to the communities most impacted within a given region. Without consulting local community members affected by the HIV epidemic, interventions crafted by pharmaceutical and state-led coalitions far removed from these communities risk being irrelevant or offensive [32-34]. An example of this occurred in 2010, when the Illinois Department of Public Health launched a campaign that stitched together the faces of 4 different men of color with the catchphrase, “He’s the one that could infect you” [35]. This fear-based campaign directly linked an entire community of black men as the primary *transmitters* of HIV—essentially vectors of disease—and stigmatized men of color as unhealthy and untrustworthy [36]. Conversely, subsequent campaigns have employed sex-affirming and culturally relevant messaging to appeal to communities impacted by HIV. In early 2016, AIDS Project Los Angeles launched *It Feels Good,* a PrEP campaign that paired its slogan with HIV prevention tips, such as “It feels good protecting myself against HIV with PrEP; It feels good discussing PrEP with my doctor; [and] It feels good telling my friends that PrEP helps protect them against HIV” [37]. The campaign tapped into concerns voiced by the LGBTQ community within Los Angeles about PrEP and leveraged them into ways to promote PrEP as an option for HIV prevention.

### PrEP4Love: #ContractHeat, #SpreadTingle, #CatchDesire #TransmitLove

In Chicago, the PrEP4Love (P4L) campaign was launched in early 2016 across the Chicago Transit Authority (CTA) through a Web-based presence with campaign ads, a campaign website and through P4L-sponsored pop-up events [31]. Although advertising PrEP as an HIV prevention tool, the campaign prioritized intimacy and desire, framing consensual sexual activities as healthy and pleasurable [38]. The sex-positive approach of P4L is a means of working toward health equity by affirming pleasure and desire in gender and sexual minority communities; the prevention campaign is uniquely risk reductive *and* sex-positive. This approach celebrates non-normative gender expressions and sexualities that are often stigmatized and marginalized. Furthermore, it builds trust between local communities and their providers, while validating diverse relationships and reinserting and encouraging sexual pleasure as a part of HIV prevention [39]. Addressing stigma and building community are the 2 unique contextual equity factors that the P4L campaign addressed. As Gilead has started using advertising in 2018, they have also adopted a sex-positive approach assumed to be inspired by campaigns such as P4L [40].

This paper aimed to address the intersection of health equity, public health, and social media marketing in the context of a public health campaign that offers PrEP as an HIV prevention strategy for underserved gender and sexual minority populations. This is done by evaluating if the P4L campaign met its primary goal of increasing PrEP awareness among black, gay, and bisexual men and other men who have sex with men and black transwomen in Chicago.

## Methods

### Campaign Development

The P4L campaign was produced by the Chicago PrEP Working Group, now significantly expanded and called the Illinois PrEP Working Group (IPWG). Initially, this group kick-started the P4L campaign through US $350K raised from a challenge grant from the Alphawood Foundation (US $250K) and private donations from individual Chicagoans (US $100K) [31]. IPWG secured pro bono creative support and volunteer hours in the summer of 2015 from a collection of Publicis Groupe advertising agencies with shops in Chicago, including Leo Burnett, Starcom, Razorfish, and Spark. Private funding and perspectives leveraged by community organizations familiar with sexual subcultures heavily affected by HIV and sexually transmitted infections enabled the IPWG to challenge and redesign the narrative on HIV prevention to one that takes seriously feelings of desire, sexual pleasure, and sexual intimacy shared between sexual actors without the use of fear as a tactic within these relationships. The sex-positive message was driven by research: A 2015 study noted one of the strongest predictors for adopting PrEP among gay men and other men who have sex with men (MSM) was the desire to increase intimacy between themselves and their partner [41], a finding that recently challenged assumptions built into some former high-profile fear-based HIV prevention campaigns [32]. Concurrently, the IPWG also sought to use the campaign as a vehicle to address health inequities in Chicago [31].

**Figure 1:**
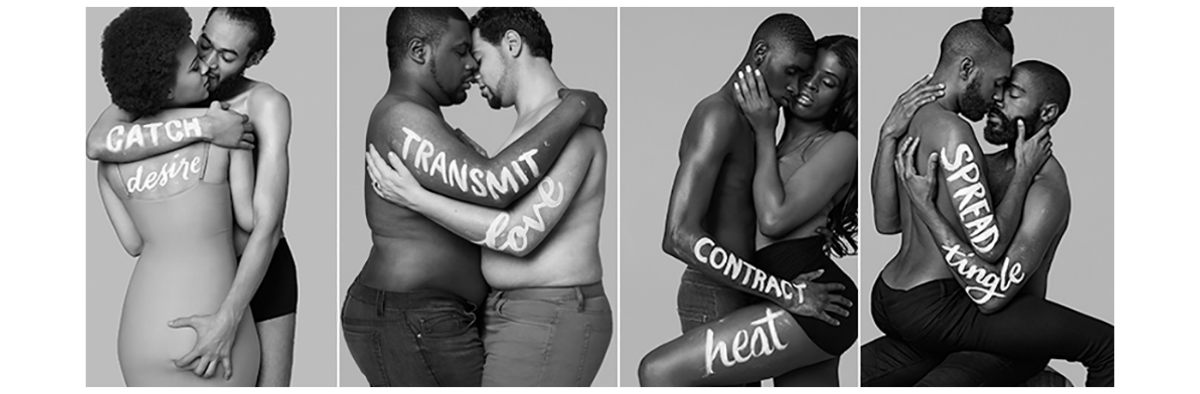
PrEP4Love advertising photos, 2016.

**Figure 2:**
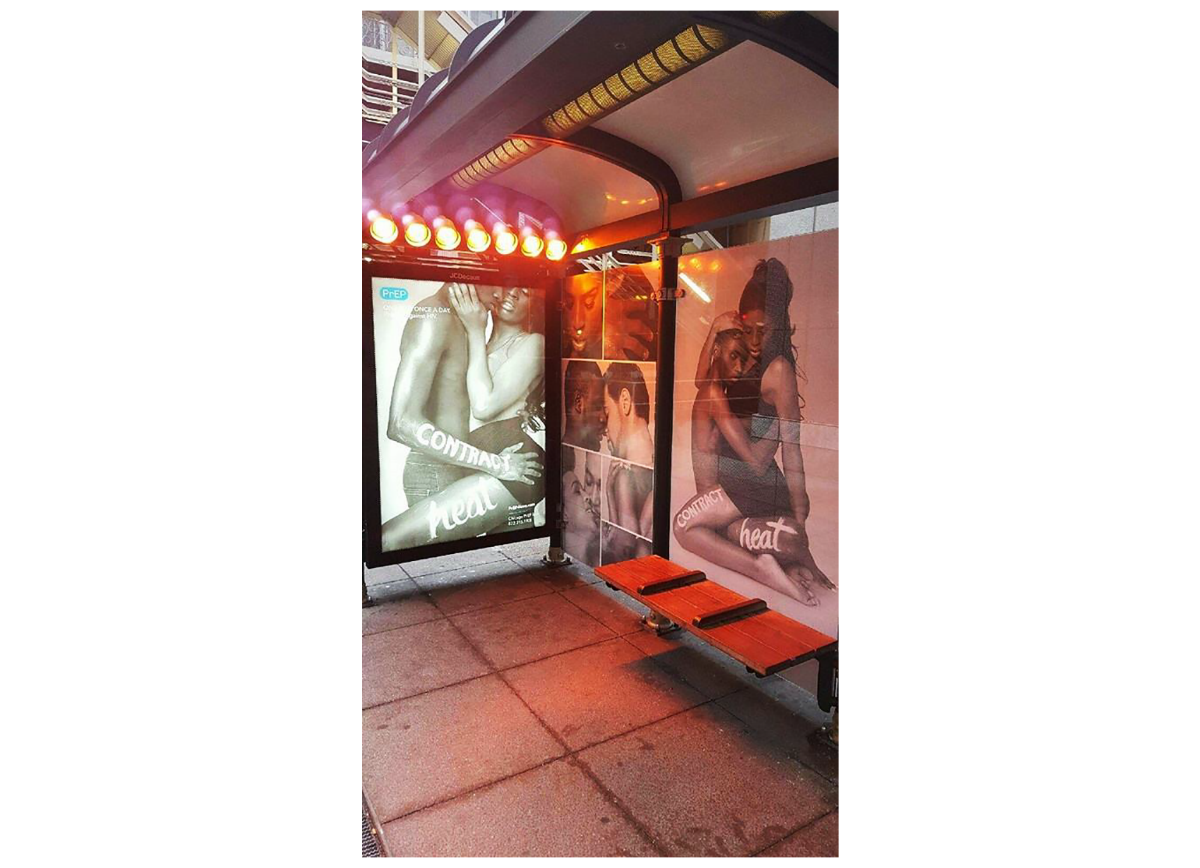
PrEP4Love campaign at a Chicago Transit Authority bus stop, Chicago, Illinois.

**Figure 3:**
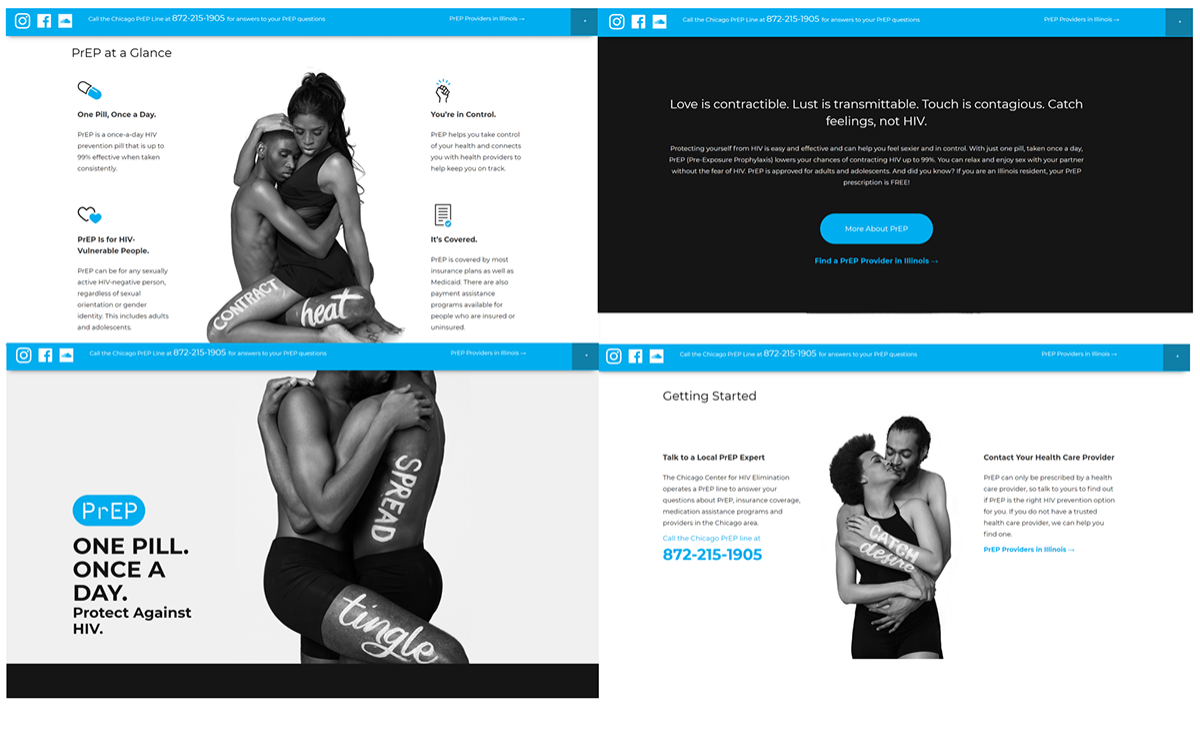
The PrEP4Love website: 4 different snapshots.

The IPWG developed culturally competent marketing materials, utilizing cross-organizational collaborations to reimagine the ways public health messaging reached vulnerable populations within the city. Developing P4L’s sex-positive HIV prevention marketing campaigns took the combined efforts of 60 creative professionals over 10 months, clocking over 1200 hours, to produce 8 final photographs and 50 plus unique files across 15 media formats [31]. The P4L campaign paired fear-based epidemiological language commonly used to describe HIV infection (eg, catch, transmit, contract, and spread) with words describing positive aspects of sex and intimacy (desire, love, heat, and tingle) [42]. Figures 1 and 2 showcase the visual advertisements, where words were painted on the bodies of 2 individuals in each other’s embrace. What makes P4L notable is its impressive digital and physical scale and reach, its ability to ally with various media marketing firms, and its capacity to tailor PrEP messaging to specific gender and sexual marginalized communities in Chicago [29]. The final photographs are simple artistic portraits of community members posed in intimate positions that represent the 3 populations most affected by new HIV infections: black, gay men and other MSM, transgender women of color, and black cisgender heterosexual women (Figures 1 to 3). Campaign content was shared with 3 focus groups of 7 to 10 participants representing each of these populations and a fourth group mixed with a proportion of the 3 target populations to assess attitudes on the campaign and to gain feedback to refine the creative content.

### Implementation

P4L Web-based advertising launched in February 2016 (Facebook, Instagram, and *smart ads*, defined as ad views that are acquired from a cohort of specific keyword Google searches) and across the CTA, including buses, train stations, platforms and cars, and gay bars using posters and drink coasters throughout Chicago. The placement of these advertisements was based on 3 specific train lines of Chicago (Orange, Green, and Red) that run through primarily black neighborhoods on the south (Green line and Red line) and west sides (Orange line) of Chicago. The red line also reaches the far north side and the *Chicago gay enclave* of *boystown* [43,44]. As P4L aimed to reach the black, gay and trans communities and because of Chicago’s racial community segregation, the campaign stakeholders decided to place advertisements along these routes to increase their reach among these populations.

The campaign advertisements directed viewers to the P4L website [45]. The P4L website (Figure 3) directs visitors to the Chicago PrEPLine, a warm-line that provides tailored information about PrEP and links visitors to PrEP services. The Chicago PrEPLine is staffed by employees of the University of Chicago who connect callers to PrEP care. This includes answering questions about PrEP, scheduling their PrEP appointments, and providing transportation services to get to scheduled appointments. The P4L website also guides visitors to a *live* list of PrEP providers in Illinois as well as the *PrEP at a glance* tool, which includes information on PrEP and insurance coverage, who PrEP is for, and how often you would need to take PrEP for it to be effective. The website also has a list of frequently asked questions and upcoming P4L pop-up events. The backdrop of the website is the P4L campaign images (shown in Figure 3).

### Data Collection

We collected the first round of user behavior data from 4 different sources: (1) the CTA website, used to report information on Chicago’s commuter traffic data and physical P4L advertisements; (2) Google Analytics, used to report information on the P4L Web-based ad campaign and the P4L website data; (3) the Chicago PrEPLine, used to report outcomes of those seeking PrEP from the campaign; and (4) the P4L pop-ups [46], used to continue the conversation of P4L and initiate and drive community-determined health care.

Chicago’s commuter traffic data were acquired through the CTA website [47]. We used the following Google Analytics metrics to acquire the remainder of our results: *impressions*, or data on P4L ad views, ad clicks (including clicks per day), *smart ad* views, ad views acquired from social media broken down between Facebook and Instagram, and lastly P4L website Web-based sessions, which includes time spent on the website, demographics of those who spent time on the website, and navigation statistics on the website. The impressions, or ad views, were the key performance indicator, as increasing PrEP awareness was the primary outcome for the campaign. To attribute website traffic to a campaign, tracking codes may be used; however, tracking codes were not used within the P4L campaign. Nevertheless, because this was a grassroots campaign effort that was only advertised on the CTA system, at local bars and clubs, and through the Web-based ads P4L allotted, we may be able to assume that website traffic was mostly attributed to the P4L campaign.

PrEPLine data are collected in the data collection tool, REDCap [48], by the staff members of the University of Chicago. Typically, a caller will call the PrEPLine number, and the PrEPLine staff assigned *on call* will answer the phone (or call back within 24 hours if missed) and answer any questions that the caller has about PrEP. The staff will also aim to link the caller to a PrEP provider if the caller indicates interest. All data, including demographics, scheduling information, and outcomes, are recorded in REDCap. PrEPLine data are cleaned monthly.

There were 2 live P4L pop-up events [46]: *Crazy Sexy Cool* that focused on young, gay, black men held on February 8, 2016, and *Formation* that focused on black and Latinx cis and transgender women held on March 9, 2016, included in our analysis. Both pop-ups were local, community-led gatherings, accompanied by live music, performers, educational material with discussion, and a P4L photo booth. There were no formal data collected at these events outside of the number of events themselves, but there was promotion of the events on the P4L Instagram and Facebook pages, as well as sponsorship for the event by P4L.

### Data Analysis

The analysis period was February through May 15, 2016. The analysis included descriptive statistics for impressions or data on P4L ad views, ad clicks (including clicks per day), *smart ad* views, ad views acquired from social media broken down between Facebook and Instagram, and lastly P4L website Web sessions. The data we received were a direct output from Google Analytics, which is tracked through the Urchin Tracking Module system (UTM). UTMs are the parameters appended to a URL that indicate how site data should be attributed per page session. These data were then sent to our team where the descriptive analyses were conducted.

The analysis methods for the PrEPLine data include running a REDCap report of those who have called the PrEPLine within the data analysis period of their demographics and outcomes.

There were no analysis methods for the pop-up events because no formal data were collected at the events.

## Results

### Physical Locations

Advertising with the CTA generated an estimated 7 million views during the month of February 2016 based on commuter flow data. P4L advertising was plastered in the interiors of 800 trains and buses for 1 month and was represented on 100 train platforms. In addition, P4L was featured in a specially branded, heated, bus shelter (emphasizing the *contract heat* message; Figure 3) in a heavily trafficked, downtown area of Chicago at the crossroads of numerous bus and rail lines. Branded beverage coasters were deployed at 16 bars across Chicago for 2 months. During this time, 7 of the 16 bars also featured print advertising in restrooms [31].

### PrEP4Love Web-based Ad Campaign

#### Views

The digital campaign coincided with the CTA launch on February 12, 2016, and lasted 2 months, finishing its first round on April 12, 2016. A total of 40,913,560 unique views were generated across various social media platforms (Table 1). Unique views indicate that the website was visited on about 41 million separate occasions, and individuals may have visited the website multiple times. A total of 32,223,987 views were received from *smart ads*, advertising attached to word searches on search engines such as Google; the 3 most clicked on ads were *STD Signs & Symptoms–More Information on STD Symptoms*, *HIV & AIDS Prevention*, and *HIV Prevention Medication*. An additional 6,970,127 views were gained through Facebook and another 1,719,446 views through Instagram.

**Table 1:** PrEP4Love ad views.

Type of views	Views, n
*Smart ad* views	32,223,987
Facebook ad views	6,970,127
Instagram ad views	1,719,446
Total views	40,913,560

#### Clicks

A total of 24,548 users clicked on P4L ads when the ads appeared on their desktops or media devices. There was an average of 182 clicks per day on the P4L website. *Click-through rate* or CTR or the number of clicks divided by impressions or views, is used to determine the rate of clicks, or interest, an ad is receiving. Industry standards for a social campaign that uses social media platforms such as Facebook or Instagram, hold a benchmark standard CTR range of 0.5% to 0.9%. P4L’s CTR was 0.0006, or 0.06%, (24,548/40,913,560); therefore when rating P4L as a social campaign, it is below industry standards.

### PrEP4Love Website

Data analysis from the P4L website started on February 1, 2016, and is ongoing through the AIDS Foundation of Chicago and the University of Chicago. As of May 15, 2016, there were 30,881 Web-based sessions among 28,485 users. Unique users accounted for 83.00% (25,632/30,881) of these sessions and 16.99% (5249/30,881) of sessions were returning users. The average time spent on the site was 55 seconds per user (range=20 seconds to 2 min 58 seconds), a rate that had steadily risen since the site launched and hovered over 1 min during the final 4 weeks of analysis. User demographics demonstrated more males (53.00%, 15,098/28,485) visiting the site compared with females (46.99%, 13,387/28,485). Interestingly, there was a higher proportion of 25- to 34-year-olds (34.00%, 9685/28,485) than 18- to 24-year-olds (18.00%, 5128/28,485) who visited the site. User behavior data revealed patterns on how users interacted with the P4L website. In total, 6.04% (1721/28,485) of Web users clicked on a link that takes them to more information about Chicago-based PrEP providers. In addition, users clicked into the *PrEP clinics and provider* link 2441 times and spent an average of 3 min on the page. *What is PrEP* has received the most user clicks (n=1732), followed by *What about condoms* (n=1556), *Side effects* (n=1459), and *Is PrEP for me* (n=1308). There may be a relationship to further investigate between campaign funding and website activity: because the advertising has stopped, the number of clicks across the site has dropped (data not shown).

### Chicago PrEPLine

An additional way of tracking those who were referred for PrEP care through the influence of P4L is to review the data from PrEPLine during the analysis period. P4L partnered with PrEPLine, a program that serves as a liaison between potential PrEP initiators and PrEP providers by linking those interested in PrEP care through phone calls, text messages, Facebook messages, and in-person encounters. PrEPLine’s warm-line number was disseminated through all P4L physical and digital marketing campaigns. PrEPLine logged 83 unduplicated encounters with participants during the analysis period. The majority of PrEPLine callers identified as African American or black, young, cisgender, gay or same gender loving men (as shown in Table 2).

During the analysis period, 51% (42/83) indicated being ready for PrEP, 25% (21/83) scheduled an appointment, and 18% (15/83) initiated PrEP. Of the 15 individuals who initiated PrEP, more than half (53%, 8/15) named hearing about PrEP through the P4L campaign. In the 83 encounters as a whole, 34% (28/83) named hearing about PrEP or PrEPLine through the P4L campaign.

**Table 2:** Sociodemographic characteristics by PrEPLine Outcome (n=83).

Demographics		Total^a^ (N=83), n (%^b^)	PrEP^c^ questions and concerns addressed (n=29), n (%)	Successfully initiated PrEP (n=15), n (%)	Lost to follow up and no longer interested in PrEP (n=39), n (%)
**Age (years)**					
	<20	6 (12)	2 (18)	0 (0)	4 (17)
	20-24	14 (29)	2 (18)	3 (20)	9 (39)
	25-29	13 (27)	4 (36)	6 (40)	3 (13)
	30-34	4 (8)	0 (0)	2 (13)	2 (9)
	35+	12 (24)	3 (27)	4 (27)	5 (22)
**Gender**					
	Cis male	42 (81)	9 (64)	13 (87)	20 (87)
	Cis female	9 (17)	5 (36)	1 (7)	3 (13)
	Other or not listed	1 (2)	0 (0)	1 (7)	0 (0)
**Race (check all that apply)**					
	Black	44 (77)	12 (70)	13 (72)	19 (86)
	Latin(x)	4 (7)	1 (6)	2 (11)	1 (5)
	White	4 (7)	1 (6)	2 (11)	1 (5)
	Don't know/other/not listed/declined	5 (9)	3 (18)	1 (5)	1 (5)
**Sexual orientation**					
	Gay or same gender loving	19 (50)	3 (50)	8 (53)	8 (47)
	Bisexual	5 (13)	0 (0)	2 (13)	3 (18)
	Heterosexual	14 (37)	3 (50)	5 (33)	6 (35)

^a^Missing data not included in table.

^b^May not sum to 100% because of rounding off.

^c^PrEP: pre-exposure prophylaxis.

## Discussion

### Major Themes

I'm just letting you know that I am sitting next to a couple at the State [and] Lake bus stop, who discussed #PrEP and praised it. And then started making out while leaning up against your picture... right next to me. My night is made and you are changing the world.

Feedback from a heated bus shelter, February 2016

Our analysis provides 3 major themes that contribute to equity-focused health research. First, we met our main objective of the campaign which was to disseminate awareness and information regarding a new biomedical HIV prevention method targeting individuals who are most at risk of contracting HIV, which was positively received by the public using a health equity approach. This was demonstrated by the reach of nearly 41 million ad views and the high volume of website engagement. P4L user behavior data collected from the website shows us that the first wave of marketing had a positive aggregate impact on PrEP awareness within Chicago through the percentage of returning site users and the increasing amount of time users spent on the website. Furthermore, the PrEP provider page was the most popular link clicked by users, where users spent an average of *3 min* per visit. This could indicate that users were seeking to connect with providers for PrEP adoption. Data reviewed from the PrEPLine further support evidence that the campaign demand has moved beyond Web-based features to in-person calls made to obtain additional PrEP-related information and PrEP initiation. Moreover, the demographics of the PrEPLine callers indicate the P4L campaign is reaching young BMSM as this was how the majority of the PrEPLine callers identified.

Second, our analysis highlights that a health social movement can successfully side-step the increasing power of commercial and market interests in shaping public health interventions in terms of reach, measured by impressions, or ad views. Medical sociologist, Peter Conrad, argues that the increasing power of private entities, such as pharmaceutical and insurance companies, has crowded out other social forces central to health care decision making (eg, physicians and local governments) and play a major role in determining how medical information and resources are currently distributed within the United States [49]. Gilead, the sole marketer of PrEP, waited *6 years* to market Truvada as a biomedical prevention option, as designated by the FDA in 2012. Activists, nonprofit community organizations, researchers, and public health departments therefore banded together to increase overall PrEP awareness around the world and to make PrEP available and accessible to populations most vulnerable to new HIV infection. The project benefited from being collaborative, collective, community-designed, and community-owned, with hundreds of members of the Chicago PrEP Working Group (now the IPWG with about 350 members).

Finally, although we successfully met our primary goal of PrEP awareness, the campaign’s CTR was below industry standards. This can happen for many reasons: the creativity of the ad did not resonate with the population that was viewing it, the ad was not reaching the intended population, the budget was not big enough to keep the *smart ads* and other campaign components running long enough, and so forth. However, based on the data from the ad views, website, and PrEPLine outcomes, we have demonstrated that grassroots campaigns, such as P4L, can be implemented successfully and reach millions of people to increase PrEP awareness using sex-positive and health equity approaches. In addition to the successes monitored through the website and ads, other research was also conducted through Northwestern University on the P4L campaign between June 2017 and April 2018 [50]. The RADAR Study, with a cohort of 700 people, those who had seen a P4L ad in Chicago (75.9%, 531.3/700) were nearly 3 times as likely to talk to their physicians about PrEP, 2 times as likely to initiate the conversation about PrEP with their physician, and nearly 2 times as likely to initiate PrEP within the last 6 months [50]. These findings contribute to the knowledge we have of the PrEP outcomes of those who have seen the P4L ads and demonstrate not only the reach of the campaign but the direct impact on PrEP uptake.

### Campaign Feasibility and Evaluation

Private funding allowed for artistic freedom in the campaign design and underlines the importance of pleasure and intimacy in HIV prevention. Sociologists such as Kane Race note the remarkable void of pleasure-based HIV prevention campaigns when it comes to harm reduction sexual health campaigns [39]. We demonstrate the feasibility of reincluding pleasure in harm reduction sexual health campaigns. Though fear-based campaigns can be effective in changing health behaviors in the short term [51], they are the subject of intense ethical debate and possibly less effective long-term than positive or gain-based public health messaging [32,33,35].

As indicated early on, the budget for the initial launch of the campaign was US $350,000. There is a direct relationship between funding and user engagements. Site activity dropped and, therefore, so did PrEP awareness reach, when sponsored advertising stopped in mid-April 2016 (data not shown). Mobile technology traffic, particularly, fell dramatically after sponsorship ended, accounting for 20.00% (8,182,712/40,913,560) of all site traffic. To have a lasting impact and a robust Web presence, public health campaigns require persistent funding to maintain various digital modalities with which users regularly engage.

This campaign is specific to Chicago in several ways: Chicago is an urban, liberal area that has the support and community to launch a sex-positive HIV prevention campaign that displays embracing partnerships of gender and sexual minorities. It should be mentioned that PrEPLine did receive a small percentage of calls that provided racist and homophobic feedback relating to the campaign and there were some instances of reported vandalism on the physical ads. This feedback, however, was not overwhelming and was used as an opportunity to educate the callers if the callers were willing to engage. There is also a commuter system in place that is able to double as an advertisement launch pad for the campaign, which was strategically placed based on the racial segregation within the city of Chicago that is mapped out by the train lines. Finally, PrEP care in Chicago is readily available and accessible. This campaign is feasible to recreate in similar urban areas but will have other conditions to consider when planning the campaign launch in other settings, for example, in the southern states or in rural areas. Some potential barriers may include access to PrEP (cost, insurance coverage, transportation, and distance), stigma, and campaign funding. However, the potential solutions to these barriers would be for primary care physicians to educate themselves and each other on PrEP for HIV prevention as a way to minimize transportation, distance and stigma as a barrier. Another way to mitigate stigma would be to offer peer change agent strategies to those who interact with the campaign for their own benefit or interest, but ultimately providing them with strategies to disseminate PrEP information to others in their network who would benefit from PrEP for HIV prevention [52]. These strategies can be offered on the campaign website and could even be integrated as part of the *PrEPLine’s*, or campaign-directed warm-line’s, discussion of *how to talk to the callers’ networks about PrEP*. Regarding the financial burden of PrEP, advocacy around Medicaid coverage for Truvada as PrEP, Gilead-funded payment programs that cover the cost of Truvada as PrEP, and working with eligible health care organizations who are registered to collaborate with the 340B Drug Discount Program, the US federal government program used by pharmacies to lower the cost of drugs significantly, [53] are all ways to provide low-cost or no-cost Truvada as PrEP for uninsured or underinsured patients.

Other important components that any city or state can implement from P4L include something similar to the PrEPLine or the information hub for those who want more information on PrEP. This information line can develop resources and systems to support those who are looking to get linked to PrEP care. The collaborative manner in how the campaign was created was also essential in its success. Groups that are constructing a sex-positive public health campaign should equally partner with community members so that the campaign is culturally competent, inclusive, and affirming. Finally, an evaluation process is important and not always conducted with public health campaigns. Yet, the evaluation component is essential for understanding whether the campaign is effectively delivering the expected outcomes. If these recommendations are implemented, there may be higher rates of success in future health campaigns that recreate a similar campaign as P4L.

### Limitations

Limitations exist in the approach of P4L as well. Although using sex-positive messaging in the campaign is intended to be empowering and was well-received by target audiences, it can also provoke stigmatizing responses. The IPWG engaged sex-positivity in its advertising campaign as a way to reintroduce the discourse of sexual pleasure into medical conversations on HIV risk and prevention. Normative understandings of gender expression and sexualities continue to impact sex-positive HIV prevention work and will subsequently shape future public health campaigns’ reach and constitution. Another limitation is that we do not possess more detailed demographic data about those who were counted among the unique views, clicks, and word searches. Even though transportation advertisements were intentionally placed in the pockets of Chicago in areas populated by people of color and therefore those most affected by HIV, it is difficult to conclude whether we effectively reached gay, black men, other black MSM, black ciswomen, and transgender women of color during our analysis period. However, Northwestern University’s data, collected between June 2017 and April 2018, do have demographic information as the demographic and P4L data were attached to their research project, RADAR [50]. This cohort (n=700) that reported having 75.9% (531.3/700) who had seen a P4L ad in Chicago, was made up of 31.7% (222/700) Hispanic or Latinx, 30.3% (212/700) white, 27% (189/700) black orAfrican American, and 11% (77/700) other. Participants in RADAR’s cohort identified as 91.6% (641/700) cisgender male, 4.3% (30/700) transgender female, and 4.1% (29/700) other [50]. The age make up was 16 to 31 years and 69.6% (487/700) identified as gay[50], 17.7% (124/700) identified as bisexual, 7% (49/700) identified as queer, 2.7% (19/700) identified as heterosexual or straight, 1.1% (8/700) identified as unsure or questioning, and 1.9 (12/700) % identified as other [50]. From these data, we can see that the campaign did reach young, BMSM, which aligns with the aim of the P4L campaign. Another limitation would be that we did not have tracking codes within the P4L website. Although we can assume that the traffic is attributed to the promotion from the campaign, we cannot be fully confident in that statement. We also did not meet industry standards for the CTR and would want to analyze this further and test in the future. With regard to PrEPLine, although we did collect and report on PrEPLine demographic data that underline PrEPLine and P4L reaching young BMSM, the total number was low and there were missing data. This was because although PrEPLine staff attempted to collect demographic information from all callers, there was less success in completing the demographics portion of the database with those who were simply calling for PrEP information instead of those who were ready to schedule a PrEP appointment. Finally, although pop-up events [46] spurred P4L activity in important ways across Chicago, the campaign did not use any data collection tools during these early on events during the analysis period. Since our analysis period, there have been 2 additional live pop-up events: *Black Joy* created for cis and transgender women held on October 4, 2016, and *Black Tea* designed for young, gay, black men on May 5, 2017.

### Future Research

Future research should gather demographic information either through increased advertising for PrEPLine or short, digital surveys when each user visits the website. Future research could also use such data to inform predictive factors of those who spent the most time on the website and those who had the most clicks, and we could potentially link individuals who completed the Web-based demographic survey to PrEPLine and PrEP outcomes. More specifically, it is essential to collect data on those who visit the P4L website to better understand how the Web-based components impact their PrEP awareness, linkage, and uptake. It is also important for public health campaigns, such as P4L to invite more young people and cisgender and transgender, black women to join the movement so as to ensure their health care experiences are culturally competent and sexually affirming. As there were no individuals who were younger than 20 years, and low numbers of cisgender and transgender, black women successfully linked to PrEP care through the PrEPLine in this analysis, more attention should be brought to these groups. This may include more activity on the P4L social media sites, but also potentially hosting youth-centered, female-centered, and transgender and gender nonconforming pop-up events. This would be a great opportunity to acquire in-person data collection that was not collected at the first 4 pop-ups (*Crazy Sexy Cool*, *Formation*, *Black Joy*, and *Black Tea*). There were also 3 focus groups that were used to inform the campaign and analyzing data collected during those focus groups may be a substantial contribution to the sex-positive public health campaign research field as well.

P4L is the first campaign of its kind in terms of reach and scale. Similar grassroots PrEP campaigns have appeared across the world in response to new understandings of HIV prevention. Since the campaign, there have been several initiatives that have been launched that continue to promote sex-positivity and *spread tingle*. New initiatives with European partners have helped shape the P4L campaign in France [54], for example, where they have adopted the P4L campaign, recreated the photos, and made videos for their website that demonstrates the same messaging as Chicago’s campaign. P4L has also been a local success with support offered from the Chicago Department of Public Health, which launched the second wave of the campaign in the fall of 2017 across the city’s transit system. AIDS Project of the Ozarks, a community-based grassroots organization housed in Springfield, Missouri, is launching the original P4L campaign in southwest Missouri. The only adaptation is the addition of their local PrEP linkage and support information. P4L is a work in progress and, because of that, there is no definitive timeline of when the campaign will end. P4L is not *done*, even during times without paid advertising, it lives through its live pop-up events and ongoing social media posts and continues to track engagement and reach in the community. The next steps for the P4L campaign are to launch a Latinx-specific component. This paper demonstrates the success and limitations of the first wave of a data-driven, sex-positive sexual health campaign that fostered respectful community engagement. Through the success of this campaign we also underscore a couple of take-away points. First, implementing P4L required a substantial amount of start-up financial capital. Second, the importance of readily available and accessible PrEP resources to which the campaign can refer inquiring and interested viewers. Finally, the success of this campaign was brought about, in part, through the relationships brokered by Jim Pickett with supportive marketing and advertising agencies. These resources, together, were important in the launch and success of the sex-positive P4L campaign and therefore central to the dissemination of PrEP health information, PrEP awareness, and accessibility of PrEP in Chicago.

## Abbreviations

BMSM: black, gay and bisexual men and other men who have sex with men
CTA: Chicago Transit Authority
CTR: click-through rate
FDA: Food and Drug Administration
IPWG: Illinois PrEP working group
LGBTQ: lesbian, gay, bisexual, transgender and queer
MSM: men who have sex with men
P4L: PrEP4Love
PrEP: pre-exposure prophylaxis
UTM: urchin tracking module
